# What did the public think of health services reform in Bangladesh? Three national community-based surveys 1999–2003

**DOI:** 10.1186/1478-4505-5-1

**Published:** 2007-02-26

**Authors:** Anne Cockcroft, Neil Andersson, Deborah Milne, Md Zakir Hossain, Enamul Karim

**Affiliations:** 1 CIETcanada, 319-1 Stewart Street, Ottawa, ON, K1N 6N5, Canada; 2 Centro de Investigación de Enfermedades Tropicales, Universidad Autónoma de Guerrero, Apdo 82, Acapulco, Mexico; 3 Planning Unit, Directorate of Health Services, Dhaka, Bangladesh; 4 Health and Life Sciences Partnership, 5-23 Old Street, London EC1V 9HL, UK

## Abstract

**Background:**

Supported by development partners, the Government of Bangladesh carried out a comprehensive reform of health services in Bangladesh between 1998 and 2003, intended to make services more responsive to public needs: the Health and Population Sector Programme (HPSP). They commissioned a series of surveys of the public, as part of evaluation of the HPSP. This article uses the survey findings to examine the changes in public opinions, use and experience of health services in the period of the HPSP.

**Methods:**

We carried out three household surveys (1999, 2000 and 2003) of a stratified random sample of 217 rural sites and 30 urban sites. Each site comprised 100–120 contiguous households. Each survey included interviews with 25,000 household respondents and managers of health facilities serving the sites, and gender-stratified focus groups in each site. We measured: household ratings of government health services; reported use of services in the preceding month; unmet need for health care; user reports of waiting times, payments, explanations of condition, availability of prescribed medicines, and satisfaction with service providers.

**Results:**

Public rating of government health services as "good" fell from 37% to 10% and the proportion using government treatment services fell from 13% to 10%. Unmet need increased from 3% to 9% of households. The proportion of visits to government facilities fell from 17% to 13%, while the proportion to unqualified practitioners rose from 52% to 60%. Satisfaction with service providers' behaviour dropped from 66% to 56%. Users were more satisfied when waiting time was shorter, prescribed medicines were available, and they received explanations of their condition.

**Conclusion:**

Services have retracted despite increased investment and the public now prefer unqualified practitioners over government services. Public opinion of government health services has deteriorated and the reforms have not specifically helped the poorest people. User satisfaction could be increased if government doctors improved their interaction with patients and if waiting times were reduced by better management of facilities.

## Background

The Bangladesh Health and Population Sector Programme (HPSP) 1998–2003 [1,2] explicitly aimed to achieve a more client-centred service, more responsive to the needs of the very poor and women. Amongst other reforms, a key element of the HPSP was unification of health and family planning services, until then covered by separate wings of the Ministry of Health and Family Welfare and operating independently on the ground. Another important aspect was construction of a large number of community clinics so that services would be provided from these clinics rather than by home visits from community health and family planning workers.

The HPSP was not fully implemented as intended. The problems are reviewed in the World Bank Implementation, Completion and Results (ICR) report for the HPSP project, in which the World Bank played a lead role in the consortium of development partners [3]. According to the ICR report, which rated the overall project outcome as 'unsatisfactory', the unification of health and family planning services had negative consequences because of the disruption it caused. Although supported at local level, unification was resisted from the beginning at district level and above. In 2001, the national government changed and the unification was halted, and reversed formally in 2003 [4]. The new government after 2001 also halted the programme of constructing community clinics and re-instituted home visits; even before this many of the clinics had not become operational as quickly as intended [5,6].

As well as undertaking internal reviews of status of performance indicators [7] and using information about health status during the period from DHS surveys [8-10], the government and development partners commissioned a series of three community-based surveys as part of the HPSP programme monitoring and evaluation. The surveys measured public perceptions and use of government services, and the satisfaction of service users, and included an analysis of factors related to better public experiences and views, as an aid to future programme planning [11-13].

A review of methods to incorporate patient views concluded that efforts to improve health care must reflect what patients want from the service [14]. Most approaches to eliciting patient views of the health services rely on contacting patients at the health facilities [15] or after they leave [16]. Contacting patients at their home addresses introduces important biases [17,18], even with reliable postal and telephone services, and is not feasible in most developing countries. Even if one could contact them all, limiting consultation to patients and ex-patients excludes the views of people who do not use the services, even though they may need them. Therefore the surveys in Bangladesh were community based and included the views of people who did not use government health services as well as those who did use them.

In this paper we draw on findings from the three surveys to review the changes in public views, use and experiences of government health services during the period of the HPSP and comment on indications from the findings about what might help to increase public use of and satisfaction with government health services in the future.

## Methods

The project, including the three community surveys described in this paper, was reviewed and approved by the CIETinternational ethical review panel prior to the first survey in 1999.

In the mid-1980s, CIET developed methods to build local measurement capacities while producing accurate and actionable data rapidly and at low cost [19,20]. The approach combines quantitative household data with coterminous data from the survey sites, including data from facilities and qualitative data from focus groups, to identify potentially effective interventions [21,22]. In Bangladesh, three survey cycles used this approach in linked samples. The first cycle in 1999 provided a baseline, while the second (2000) and third (2003) updated information on the public use, perception and experiences of health and family planning services. The Ministry of Health and Family Welfare, the bureau of statistics and local researchers participated in the design of the surveys.

A two stage stratified random sample of villages was selected within each of the six divisions, with the number of villages proportional to the population of the division. In each of the 44 selected *upazilas (*administrative areas of 2–300,000 people) we selected five (in three cases, four) villages randomly, giving a total of 217 villages. In each village, we selected 100–120 contiguous households radiating from a random starting point. A further random sample of 30 enumeration areas was drawn from the main cities in the country, the number in each city proportional to the population. The 2000 and 2003 surveys used the 1999 sample, but with a random 25% of the rural sites reselected.

The household questionnaire asked about perceptions, use and experience of health and family planning services from different providers. It was translated into Bengali, pre-tested and adjusted. For each cycle, we trained 15 teams of interviewers in four regional centres. Divisional coordinators monitored the fieldwork. The field teams also visited the *upazila *health facilities serving each sample community and interviewed the heads of the facilities, collecting information about the medicines, amenities, and equipment available, and the way the facilities were run. Following entry and preliminary analysis of the data from households, trained field teams returned to the survey sites to discuss findings in gender stratified focus groups.

We used Epi Info [23] for double data entry and validation. We defined the poorest households as those below the 25th percentile of reported household income. Unmet need for health care was a household reporting at least one member ill in the last month but having no contact with any health service in this time. We examined univariate associations, calculating the Odds Ratio and 95% confidence interval, and examined the simultaneous effects of variables by logistic regression [24], from which we derived adjusted Odds Ratios and calculated adjusted Risk Differences.

## Results

Some 47% of the household heads were literate in 1999 and 2000, increasing to 50% in 2003. Most household respondents were female in all three surveys (22,350/25,490 or 88% in 2003).

### Household opinions of government and private health and family planning services

The proportion rating the services as 'good' fell from 39% in 1999 to 10% in 2000 (OR 5.74, 95%CI 5.47–6.03; 9,836/25,518 in 1999, compared with 2,467/25,053 in 2000) and remained about the same in 2003.

We examined possible causes for the decline in public opinion in a multivariate model (Table 1). Sex and economic status of the respondent were related to rating in each cycle but they did not explain deterioration in the rating between cycles. The decline in opinion was less marked in households who reported use of the services in 2003 and in households with an illiterate head. It was also less marked in communities where the review of the local health facility revealed it to be more "user-friendly" (for example had screens for privacy or had toilets for women) and in communities where the health facility head reported there was an active health services development committee involving the public. However, most of the difference in public opinion between 1999 and 2003 was not explained by the factors examined.

**Table 1:** Final model of factors influencing general opinion of respondents, contrasting 1999 with 2003

	Crude OR	Adjusted OR	95%CI adjusted OR	χ^2^mh
Head of household illiterate 2003	0.89	0.83	0.78–0.89	26.9
Someone in household used govt health in last month 2003	0.83	0.92	0.85–0.99	4.1
Either UHC or UHFWC had curtains for examinations 1999	0.69	0.66	0.61–0.73	76.85
Either UHC or UHFWC had separate toilet for women 1999	0.78	0.77	0.68–0.87	17.36
Upazila health service development committee present 2003	1.01	0.89	0.82–0.96	10.08
Unexplained difference between 1999–2003	4.95	4.97	4.62–5.34	1000.1

OR = Odds Ratio

UHC = Upazila health centre

UHFWC = Union health and family welfare centre (more local facility).

Rating of government services, when few households use these services themselves (see below) was probably influenced by past experience, hearsay evidence from friends and neighbours, and media coverage. There was some change in the pattern of perceived problems with the services across the three cycles (Table 2). Lack of medicines was the most common complaint throughout. Bad attitude of staff was more commonly mentioned in 2000 and 2003, as were extra payments to doctors. Focus group participants echoed these complaints from the household respondents and described them more fully, making it clear that government health services had a very bad reputation with the public.

**Table 2:** Identified problems in government health and family planning services

	% (No) of respondents		
Identified problems	1999	2000	2003
Lack of/poor quality of medicines	54 (14,128)	58 (14,621)	55 (14,052)
Bad staff attitude	15 (4,015)	25 (6,276)	29 (7,447)
Bad service	29 (7,561)	40 (10,098)	27 (6,751)
Extra payment to doctors	9 (2,376)	12 (3,091)	17(4,440)
Have to pay for medicines	9 (2,202)	17 (4,263)	17 (4,379)
Difficult to reach	22 (5,588)	19 (4,785)	16 (4,164)
Doctors not available	7 (1,876)	13 (3,178)	15 (3,887)
Dirty, poor equipment/facilities	15 (3,984)	13 (3,399)	11 (2,887)
Lack of doctors/specialists/nurses	18 (4,698)	14 (3,561)	10 (2,618)
Lack of different services	6 (1,481)	14 (3,478)	10 (2,600)
No problem	6 (1,679)	1 (238)	1 (338)
Too few beds/lack of facilities	7 (1,833)	7 (1,886)	7 (1,706)

### Use of health and family planning services

Only 15% of visits to health and family planning services were for preventive purposes and these are not considered further here.

Between 1999 and 2003 there was a decrease from 13% to 10% in the proportion of households who reported at least one member using government health services for treatment in the preceding month (3,405/26,207 in 1999 compared with 2,516/25,487 in 2003; OR 1.36, 95%CI 1.29–1.44). During the same period there was an increase from 30% to 49% in the proportion of households who reported using private services (including unqualified practitioners) for treatment in the preceding month (7,752/26,158 in 1999 compared with 12,574/25,488 in 2003; OR 0.43, 95%CI 0.42–0.45).

Focus group participants gave insights into why people choose alternatives to government health services, with comments such as *"most of the time we don't find any doctors in government facilities, so we turn to the village doctors" *and *"the village doctors would come at midnight if they are called"*.

In 1999 3% (778/26158) of households had unmet need for health care. Unmet need was higher in 2000 at 11% (2892/25468) and in 2003 at 9% (2406/25475), with a significant decrease between 1999 and 2003 (OR 3.40, 95%CI 3.13–3.70). Both the 1999 and 2003 surveys were conducted in January-February, so seasonal variation cannot explain the increase in unmet need. In 2003, unmet need was more likely if the household head was female or illiterate, in rural sites and in the poorest households. These personal variables remained in a multivariate model of unmet need in 1999 and 2003, but did not explain much of the increase in unmet need between 1999 and 2003 (Table 3).

**Table 3:** Actionable factors influencing report of unmet need (households with someone ill who did not seek medical attention) in 1999 and 2003 (n51633)

	Crude OR	Adjusted OR	95%CI adjusted OR	Adjusted gain/1000	95%CI gain
Illiterate head of household	1.43	1.35	1.25–1.46	12.7	9.56–15.9
Female headed household	1.87	1.50	1.34–1.69	2.4	1.7–3.1
Rural resident	1.56	1.39	1.22–1.59	9.2	5.5–13.0
Poorer household (lowest 25 percentile 2003)	0.96	1.14	1.04–1.24	2.0	0.7–3.4
Unexplained difference between 2003 and 1999	3.41	3.48	3.2–3.77	36.8	34.5–39.2

1. The individual benefit is the adjusted Odds Ratio from logistic regression.

2. The PRI (proportion requiring intervention) is the proportion of service users who currently do not have the favourable value of the variable. For example, the proportion that currently does not get all the prescribed medicines is 80%.

3. The gain per 1000 is calculated by multiplying the PRI by the risk difference. This is the proportion who could potentially become satisfied with the service as a result of each intervention.

### Experience and satisfaction of users of health services

Among reported outpatient visits (there were less than 5% admissions) to health services for treatment in the preceding month, the share going to government facilities fell from 17% (2,575/14,614) in 2000 to 13% (2,231/17,514) in 2003 (OR 1.44, 96%CI 1.36–1.54), while the share using unqualified practitioners rose from 52% (7,633/14,614) to 60% (10,564/17,514) (OR 0.72, 95%CI 0.69–0.75). Most (43% of all visits) of the unqualified (non-medically qualified) practitioners were village doctors or "quacks", many of whom had received some paramedical training.

Between 2000 and 2003 there was a significant decrease from 66% (1,591/2,415) to 56% (1,257/2,230) in the proportion of users of government services who were satisfied with the way the service providers behaved towards them (OR 1.49, 95%CI 1.32–1.69). Satisfaction with the behaviour of private and unqualified providers was higher (over 90%) and did not change between 2000 and 2003.

Patients were much more likely to be satisfied with private or unqualified providers than with government providers in 2003 (OR 7.39, 95%CI 6.67–8.18).

Half of government service users in 2000 (50%; 1,196/2,411) felt they had a full explanation of their condition compared with 44% (981/2,230) in 2003 (OR 1.25, 95%CI 1.11–1.41). In 2003, service users who considered they had a full explanation of their condition were much more likely to report satisfaction with the behaviour of the service provider (85%, 809/981) compared with those who did not consider they had a full explanation (36%, 448/1,249) (OR 8.29, 95%CI 6.78–10.14). This association was mainly among users from households with an income at or above the 25^th ^percentile: 84% (661/787) of those with an explanation were satisfied compared with 36% (330/917) of those without an explanation (OR 9.3, 95%CI 7.32–11.9).

One third of government service users in 1999 (33%, 866/2,641) reported all the prescribed medicines were available to them from the facility compared with only 23% (435/1920) in 2003 (OR 1.67, 95%CI 1.45–1.91). In 2003, service users who got all the prescribed medicines from the facility were more likely to be satisfied with the behaviour of the service provider (79%; 343/435) compared with those who did not get all the prescribed medicines (53%; 783/1,485) (OR 3.34, 95%CI 2.57–4.35). Focus group participants blamed health workers for the failure of government facilities to provide the prescribed medicines, with comments such as *"the health workers only give medicines to known people; they don't give them to the poor" *and *"the government supplies medicine to the facilities, but we don't get them."*

Waiting times in government facilities changed little between 2000 and 2003 (median 30 minutes). In 2003, service users who waited less than 30 minutes to be attended were more likely to be satisfied with the behaviour of the service provider (59%; 875/1,475) compared with those who waited longer (50%; 382/771) (OR 1.53, 95%CI 1.28–1.84).

Around 20% of government service users reported making unofficial direct payments to service workers, with little change in this proportion over time (21%, 20%, and 18% in 1999, 2000 and 2003 respectively). Making these payments made no difference to reported satisfaction with the behaviour of the service providers.

We examined the factors associated with satisfaction with government service providers in a multivariate model to examine the reduction in satisfaction between 2000 and 2003. The model excludes the small group of users who made no payment at all for their visit. Table 4 shows the theoretical impact of strategies to improve satisfaction. Some decline in satisfaction remains to be explained (bottom row in Table 4), but there is a sizeable potential impact from changing the way doctors interact with patients, reducing waiting times, and making all prescribed medicines available.

**Table 4:** Actionable factors influencing satisfaction with behaviour of health workers, among those who used services in 2000 and 2003: Gains from different strategies (n = 4128)

	Crude OR	Adjusted OR	95%CI adjusted OR	Adjusted gain/1000	95%CI gain
Illiterate head of household	0.59	0.72	0.61–0.84	2.4	1.3–3.5
Waiting time under 20 minutes	2.11	1.81	1.53–2.14	61.4	44.1–78.6
Received all prescribed drugs	3.73	2.29	1.84–2.85	96.3	70.8–121.8
Received explanation about illness	10.41	2.89	2.3–3.63	91.6	72.1–111.2
Received explanation about remedy	10.7	4.61	3.37–5.69	154.8	133.5–176.2
Unexplained difference between 2003 and 2000	1.54	1.81	1.54–2.11	34.8	25.6–44.1

1. The individual benefit is the adjusted Odds Ratio from logistic regression.

2. The PRI (proportion requiring intervention) is the proportion of service users who currently do not have the favourable value of the variable. For example, the proportion that currently does not get all the prescribed medicines is 80%.

3. The gain per 1000 is calculated by multiplying the PRI by the risk difference. This is the proportion who could potentially become satisfied with the service as a result of each intervention.

## Discussion

Satisfaction is a capacious concept, heavily conditioned by culture, expectations and sense of entitlement [25,26]. Most people in Bangladesh have fairly low expectations of their health services [27]. Given that the survey was repeated in the same places between 1999 and 2003, "cultural" influences on satisfaction are likely to have remained more or less constant, so reductions in reported satisfaction over this relatively short period are a legitimate cause for concern. However, we recognise that extraneous factors such as political unrest might have undermined trust in public services generally during the period.

Increasing dissatisfaction of public and service users was accompanied by decreasing use of government health services and increase in the unmet need for health care. Taken together, this represents effective retraction of the health services, despite the large financial investment in the HPSP (over US $ 350 million from development partners between 1999 and 2005 [3]). This retraction seems to have been felt disproportionately by the vulnerable; households headed by illiterate and female heads, those in rural areas and those in the lowest 25% of income all were more likely to have unmet need in 2003 compared with 1999.

The three surveys reported here agree with other studies from Bangladesh. Lack of medicines in government health facilities was a frequent complaint of service users in Dhaka [28]. The 2001 Bangladesh Health and Demographic Survey [8] reported 50% of people used an unqualified doctor for treatment of illness, 17% used a private pharmacy, and only 13% saw a doctor in a government facility. A 2003 survey of government health facilities reported many unfilled posts for service providers and that many doctors were absent from the facilities at the time of unannounced visits [29].

It would be misleading to suggest that any single or simple intervention will reverse this complex situation. The surveys did offer indications of the possible magnitude of benefits of interventions to improve satisfaction of service users. A low cost intervention to improve user satisfaction would be to give patients explanations of their condition and treatment. The challenge is to persuade government doctors to talk to patients. The doctors can do this, given the right environment: in most rural areas, the government doctors are the same individuals that, seen privately, patients are mostly satisfied with and feel they have had an explanation from.

Improved management, including filling vacant posts and ensuring doctors and other service providers attend regularly, could reduce waiting times. The cost of ensuring regular medical attendance would depend on the measures adopted to achieve it. Increasing salaries would be expensive. Ensuring all prescribed medicines are available would increase the number of satisfied users, but it would be costly and is a longer-term initiative.

Improving the experience of service users may help to reverse the drift away from government services. When asked how they would persuade people to use government health services, focus group participants explained that once the quality improved, people would naturally use the services: "Why do people come to a tea stall? Because the shopkeeper behaves well and the tea tastes good."

## Conclusion

From the point of view of the public, government health services retracted during the period of the HPSP despite increased investment and the public now prefer unqualified practitioners over government services. Public opinion of government health services has deteriorated and the reforms have not specifically helped the poorest people. Analysis based on the survey findings suggests that user satisfaction with government health services could be increased relatively quickly if government doctors improved their interaction with patients and if waiting times were reduced, for example by better management of facilities.

## Competing interests

The author(s) declare that they have no competing interests.

## Authors' contributions

AC helped design the study, led the implementation of all three surveys, undertook the analysis, and drafted this manuscript. NA designed the study, advised on analysis, and reviewed the draft manuscript. DM, ZH and EK supported implementation of the surveys and helped with analysis. All authors read and approved the final manuscript.
